# The relationship between response inhibition and posttraumatic stress symptom clusters in adolescent earthquake survivors: An event-related potential study

**DOI:** 10.1038/srep08844

**Published:** 2015-03-05

**Authors:** Jianhui Wu, Yiran Yuan, Chengqi Cao, Kan Zhang, Li Wang, Liang Zhang

**Affiliations:** 1 Key Laboratory of Behavioral Science, Institute of Psychology, Chinese Academy of Sciences, Beijing, 100101, China; 2 University of the Chinese Academy of Sciences, Beijing, 100049, China; 3 Key Laboratory of Mental Health, Institute of Psychology, Chinese Academy of Sciences, Beijing, 100101, China

## Abstract

Posttraumatic stress disorder (PTSD) patients experience impaired response inhibition. Little is known about the relationship between response inhibition abnormalities and distinct PTSD symptom clusters. This study investigated the relationship between response inhibition processing and a five-factor model of posttraumatic stress symptomatology in adolescents. The event-related potentials of 54 unmedicated adolescent earthquake survivors (age 15–18 years) were recorded as they completed a Go/NoGo task. The PTSD Checklist-Specific Stressor Version (PCL-S) was used to assess PTSD symptoms. Regression analyses were conducted to examine the associations between the five symptom-cluster model and response inhibition processing. The results revealed that the avoidance symptom cluster score, but not the numbing or other clusters' scores, was positively associated with NoGo-P3 latency. These results suggest that a specific PTSD symptom cluster—avoidance—has a distinct association with the slowed speed of the late step of response inhibition processing, i.e., decision or success of response inhibition in adolescent earthquake survivors.

In the Diagnostic and Statistical Manual of Mental Disorders, 4th Edition (DSM-IV)^1^, posttraumatic stress disorder (PTSD) is defined as a severe and complex mental disorder precipitated by exposure to extraordinarily stressful events. It is characterized by marked re-experiencing, avoidance/emotional numbing, and hyperarousal symptoms. The fifth edition was published in May, 2013 and the most notable change from DSM-IV to DSM-5 is that numbing symptoms are separated from avoidance symptoms to form a new cluster of “negative alterations in cognition and mood”^2^. Although PTSD is primarily classified as an anxiety disorder, research has demonstrated that it also involves deficits in cognitive functions in both adult and youth individuals, such as emotion, attention, memory, and executive function^3^,^4^,^5^,^6^,^7^.

Response inhibition, the inhibition of prepared or prepotent responses, is one of the core components of executive function and has been found to be associated with a variety of psychiatric disorders^8^. Response inhibition has significant implications for the maintenance and treatment of PTSD^9^. Some evidence indicates that response inhibition is behaviorally impaired in PTSD patients compared with non-PTSD controls^10^,^11^,^12^,^13^. Decreased inhibitory performance in PTSD patients was positively associated with symptom severity^10^,^11^. The results of other studies, however, have not confirmed this decreased inhibitory performance^14^. Moreover, functional magnetic resonance imaging studies have found reduced activation of inhibition-relevant brain areas (i.e., the frontal cortex) in PTSD patients^10^,^15^,^16^, and greater activation of inhibition-relevant brain areas was associated with diminished PTSD severity^10^.

Previous studies generally focused on the response inhibition function under PTSD in adults. The response inhibition function for adolescents, however, might have more implications. Adolescence is a period of increased vulnerability to psychiatric problems. For one thing, adolescents are at a greater risk of experiencing trauma than either adults or children^17^. For another thing, although the brain is undergoing major remodeling during adolescence, the frontal lobe (the executive center of the brain) is the last brain region to mature in humans, and its maturation is not complete in the adolescent^18^,^19^. Using fMRI, a previous study suggested that although youth (10–16 years) who experienced trauma and had posttraumatic stress symptoms (PTSS) had a similar behavioral performance during response-inhibition tasks compared with the control youth, PTSS subjects had altered brain activity, such as reduced middle frontal cortex activation and greater medial frontal activation when compared with control subjects^6^.

Event-related brain potential (ERP), with its excellent temporal resolution, can be used to distinguish and identify the neural sub-processes involved in complex cognitive functions, such as response inhibition. One of the most common measures of response inhibition is the Go/NoGo task. Two major ERP components, frontocentral N2 and frontocentral P3, are associated with different phases of response inhibition processing. The N2 is a negative component elicited approximately 300 ms post-NoGo stimulus onset and may represent an earlier step of response inhibition, i.e., the detection of the conflict between the internal representation of the Go response and the NoGo stimulus^20^,^21^,^22^. The frontocentral P3 is a positive component elicited approximately 400 ms post-NoGo stimulus onset and may represent a later step of response inhibition, i.e., response evaluation/decision or response inhibition success^22^,^23^. One study found that the PTSD group had a longer NoGo-P3 latency than the control group^14^, but another study found a shorter NoGo-N2 latency in the PTSD group^12^.

PTSD is a highly heterogeneous clinical syndrome composed of distinct symptom clusters, which may explain the complex effect of PTSD on response inhibition. In the DSM-IV^1^, PTSD symptoms are categorized into three clusters: re-experiencing, effortful avoidance and emotional numbing, and hyperarousal. Based on this tripartite phenotypic model, a previous study reported that re-experiencing was the strongest predictor of performance on the Go/NoGo task (rho = 0.54)^9^. Another study used a youth sample (ages 10 to 16 years), and the results showed that activation in the right insula was significantly positively correlated with the avoidance/numbing and hyperarousal symptoms scores but not the re-experiencing score^6^. Javanbakht and his colleagues proposed in their review that earlier steps of information processing are usually bottom-up and more automatic and might thus be “clinically correlated with ‘re-experiencing' and ‘hyperarousal'”, whereas the latter steps of information processing are usually top-down and might be “clinically related with volitional ‘avoidance' symptoms”^24^. For example, P50 gating correlated negatively with PTSD subjects' re-experiencing intensity scores^25^, whereas the amplitude/latency of the central-parietal P300, which was usually elicited in oddball paradigm reflecting the categorization of the target, is correlated with the intensity of avoidance symptoms^26^,^27^,^28^. However, we still have little knowledge about the relationship between distinct PTSD symptom clusters and the different steps of neural processing of response inhibition in PTSD, especially in adolescents.

It should be noted that during the past two decades, many confirmatory factor analysis (CFA) studies have demonstrated that the tripartite DSM-IV model of PTSD consistently fails to capture the latent structure of PTSD symptoms^29^,^30^. The latest development in the CFA literature on PTSD suggests that a five-factor model comprised of re-experiencing (B1–B5), avoidance (C1–C2), emotional numbing (C3–C7), dysphoric arousal (D1–D3), and anxious arousal (D4–D5) provides a significantly better representation of PTSD symptoms than the DSM-IV tripartite model and two alternative four-factor models^31^,^32^,^33^,^34^,^35^,^36^. Recent studies have suggested that heterogeneous clusters of the five-factor PTSD model are associated, to varying degrees, with the biological markers of this disorder such as plasma cortisol^37^, serotonin transporter 5-HTTLPR genotype^38^ and in vivo norepinephrine transporter availability in the locus coeruleus^39^. These findings lend additional support to the newly refined five-factor model of PTSD symptoms.

The current study investigated the relationship between response inhibition and the severity of total PTSD symptoms and the relationship between response inhibition and each of the five-symptom clusters in a sample of adolescent Chinese earthquake victims. We included all participants, not just probable PTSD cases, in the final analysis. Taxometric studies support a dimensional model rather than a categorical model of PTSD^40^,^41^. An analysis of the full range of symptom severity is appropriate and informative and can also lead to higher statistical power and less bias in parameter estimation. Response inhibition was measured using a classic Go/NoGo task. The analysis focused on both behavioral results and ERP measures, i.e., the latency and amplitude of NoGo-N2 and NoGo-P3. We predicted that the total score on the PTSD Checklist would be negatively associated with the amplitude of NoGo-N2 and/or NoGo-P3. More importantly, we also predicted that several of the five-symptom clusters would be associated with the ERP measures, including the amplitude and latency of NoGo-N2/P3. According to Javanbakht *et al*.'s proposals^24^, we predicted that re-experiencing, dysphoric arousal and/or anxious arousal are associated with altered N2, the early step of response inhibition, whereas avoidance and/or emotional numbing are associated with altered P3, the late step of response inhibition.

## Results

### Descriptive analyses

Table 1 shows the means and standard deviations of the behavioral performance and PCL scores (n = 54). As suggested by previous studies^42^,^43^, a score of 44 on the PCL was used as a clinical cutoff to screen “probable PTSD cases.” According to this criterion, 23 (42.6%) participants in the sample were identified as probable PTSD cases. The mean trauma exposure score was 2.8 ± 1.4 (range: 0–6). The HSCL-25 anxiety score was 19.5 ± 5.9 (range: 10–35), and the depression score was 30.1 ± 8.2 (range: 15–51).

### NoGo vs. Go condition

**Figure 1:**
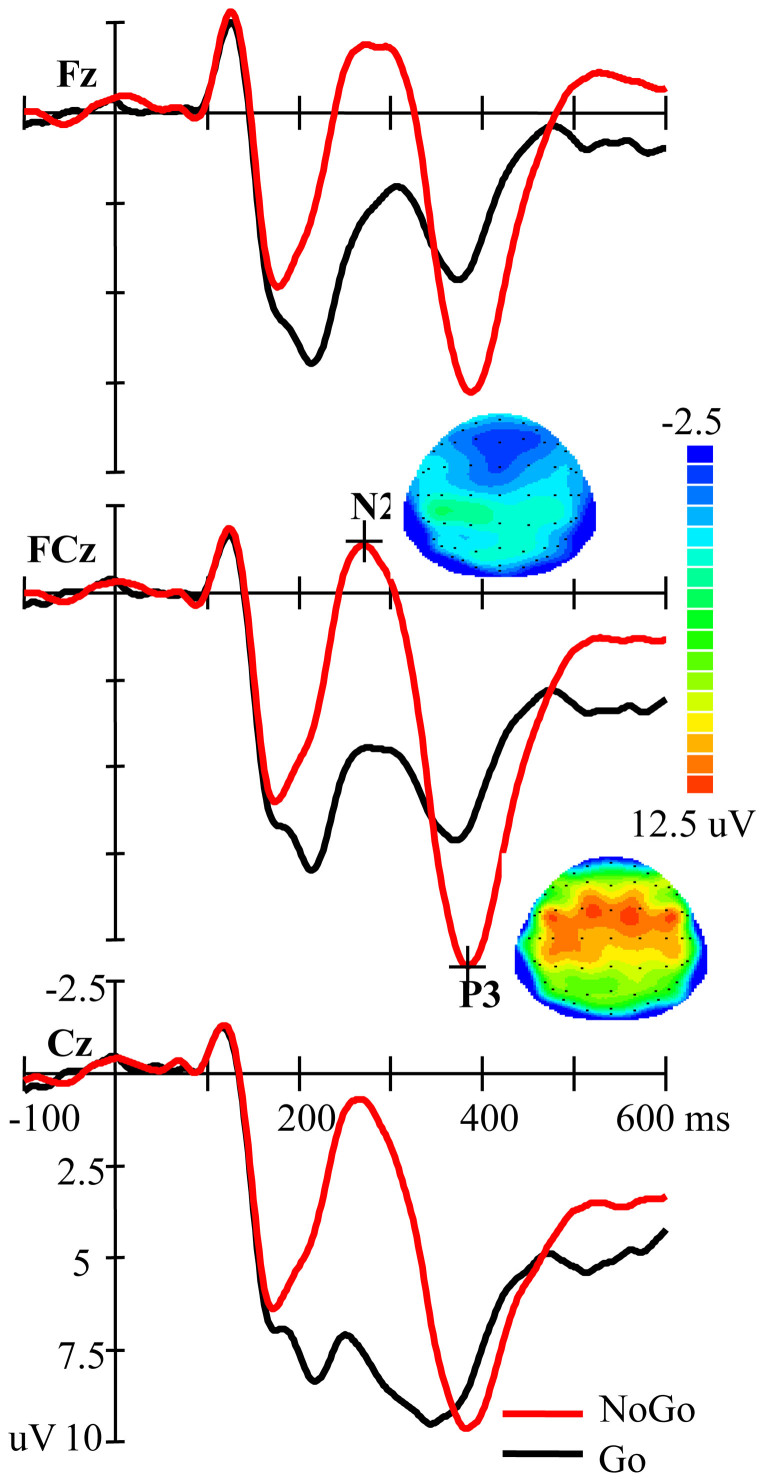
Grand average ERP for the NoGo and Go conditions at Fz, FCz, and CZ (n = 54). The scalp distributions are time-locked to the peak amplitude of the NoGo-N2/P3.

As illustrated in Figure 1, the N2 amplitude was larger for NoGo than for Go stimuli (F(1,53) = 55.551, *p* < 0.001). For the P3 amplitude, the main effect of Go-NoGo did not achieve significance (F(1,53) = 1.479, *p* = 0.229), but the Go-NoGo and site factors showed a significant interaction (F(2,106) = 39.432, *p* < 0.001, Epsilon = 0.754). A further *t*-test analysis showed that at Fz and FCz (*p*s = 0.013), but not at Cz (*p* = 0.078), the NoGo condition elicited a significantly higher P3 than the Go condition.

### Regression analyses

The preliminary correlation analysis showed that gender was significantly associated with a false alarm rate (r = 0.328, *p* = 0.016) and miss rate (r = 0.284, *p* = 0.037). No other significant associations were observed between “demographic variables and clinical variables” and “other behavioral and ERP measures” (*ps* > 0.05). Only the variables that had a significant association with the dependent variables were treated as covariates in the regression analyses.

**Figure 2:**
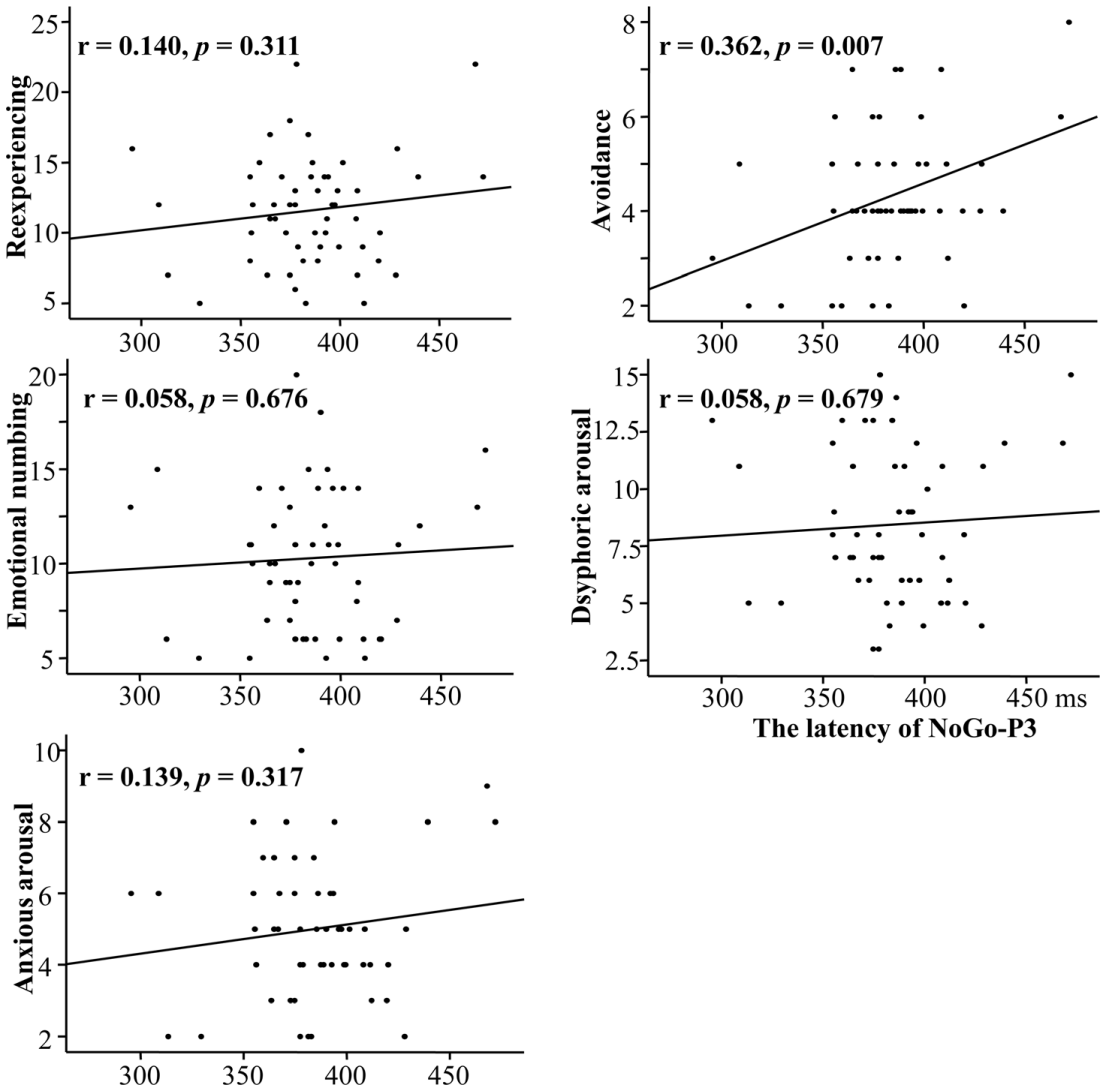
The correlation analysis scatter plot of NoGo-P3 latency (averaged across Fz, FCz, and Cz) for each of the five PTSD symptom clusters (n = 54).

Bivariate regression analyses showed a marginally significantly negative association between total PCL score and NoGo-P3 amplitude (r = −0.232, *p* = 0.092). There were no significant associations between the total PCL score and any of the behavioral indexes, NoGo-P3 latency, NoGo-N2 latency and amplitude (*p*s > 0.10). Bivariate regression analyses between each of the five PTSD symptom clusters and the behavioral/ERP indexes revealed that only avoidance had a significant association with NoGo-P3 latency (r = 0.362, p = 0.007; see Table 2 and the scatter plot in Figure 2).

Multivariate regression analyses showed that the five-cluster model predicted 16.3% of the NoGo-P3 latency (R^2^ = 0.163, F(5,48) = 1.868, *p* = 0.118). Only avoidance had a significantly positive association with NoGo-P3 latency (t = 2.699, *p* = 0.010) (see Table 2). The results from the preliminary correlation analysis showed that none of demographic variables (i.e., age and gender) or clinical variables (i.e., trauma exposure, depression, and anxiety) were significantly correlated with NoGo-P3 latency, thus not included in this multivariate regression analysis. The posthoc multivariate regression analysis showed that one avoidance symptom (C1) was significantly associated with the latency of NoGo-P3 (ß = 0.412, t = 2.46, *p* = 0.018), but that C2 was not (ß = 0.050, t = 0.281, *p* = 0.78). Collinearity statistics of the multivariate regression model are also shown in Table 2, and the results indicated that there were no explicit multicollinearity problem.

The regression analyses showed that none of the symptom clusters were significantly associated with any behavioral data, NoGo-P3 amplitude, NoGo-N2 amplitude and latency (*p*s > 0.05).

## Discussion

The current study investigated the relationship between response inhibition and posttraumatic stress symptom clusters using an ERP study. Both the multivariate regression and the bivariate correlation analyses revealed that only avoidance symptoms, not numbing or other symptom clusters, were associated with increased NoGo-P3 latency.

The results indicated that only NoGo-P3 amplitude was marginally negatively associated with total PCL score. The literature has generally considered NoGo-P3 to be an index of a later stage in the inhibitory response process, i.e., the response evaluation/decision or successful inhibition of a response^22^,^23^. This result in the present study is consistent with the literature that suggests that PTSD symptom severity is negatively associated with inhibitory performance^10^,^11^ and the activation of inhibition-relevant brain areas^10^. Our ERP study, with its excellent temporal resolution, indicated that the association between PTSD symptom severity and response inhibition occurs at a late step in the response inhibition processing, i.e., response evaluation/decision^22^,^23^.

The PTSD phenotype was composed of different types of symptom clusters. To reduce heterogeneity and increase the probability of identifying the distinct contributions of specific biological mechanisms, an alternative approach with more homogeneous symptom clusters as alternative phenotypes has been used in PTSD studies. The use of homogeneous symptom clusters for mental disorders is consistent with the Research Domain Criteria project (RDoC) initiative proposed by the National Institute of Mental Health (NIMH)^44^ and is becoming increasingly common in the field. Researchers have demonstrated that the symptom clusters of the five-factor model, a contemporary phenotypic model of posttraumatic stress symptomatology, exhibit distinct relationships with external psychopathological variables and biological processes^33^,^37^,^38^,^39^,^45^,^46^. For example, emotional numbing has been associated with cortisol^37^ and anxious arousal with norepinephrine transporter availability in the locus coeruleus^39^.

Following this approach, we further examined the relationship between response inhibition and the posttraumatic stress symptom clusters of the five-factor model. The results revealed that, of the five clusters, only avoidance was associated with an ERP measure; as avoidance scores increased, NoGo-P3 latency increased. It is worth noting that response inhibition has the false alarm rate as the behavioral index of its ability, there is no behavioral index to reflect the speed of response inhibition. The ERP technique, with its high temporal resolution, provides the neural index for the speed of response inhibition, i.e., the P300 latency. This study's results suggest that it takes more time to inhibit the response trend with a greater level of avoidance. According to the DSM, avoidance symptoms include strategic, conscious efforts to actively curtail trauma-related thoughts or feelings^45^. This effortful avoidance may delay the conscious and later stage of response inhibition, as suggested by the increased NoGo-P3 latency. Furthermore, among these five symptom clusters, only avoidance has been clearly considered including conscious efforts, which may explain why only the avoidance symptoms have a relationship with the similarly conscious sub-process during response inhibition.

This result and its interpretation echo the hypothesis of Javanbakht and his colleagues^24^, who proposed that the latter and conscious stages of information processing could be clinically related to conscious and effortful avoidance symptoms. The literature has provided evidence for this proposal, e.g., an altered central-parietal P300 has been associated with the intensity of the avoidance symptom^26^,^27^,^28^. The P300 in these previous studies generally reflects the categorization mechanism of the target stimulus elicited in the oddball paradigm. Our results suggested that the speed of the response inhibition, as reflected by the latency of front-central NoGo-P3, was associated with the intensity of the avoidance symptom. Given that avoidance symptoms are also shared by several anxiety disorders^47^, it's an interesting topic to investigate whether a similar association between avoidance symptoms and response inhibition could be observed in other anxiety disorders in future studies.

In the DSM-IV, PTSD avoidance and emotional numbing symptoms are grouped together, by expert consensus, in a single symptom cluster (i.e., criterion C); however, the DSM-IV clusters have been widely criticized, and many studies have suggested that a distinction should be made between avoidance and numbing symptoms^45^,^48^,^49^. In the DSM-5, the PTSD criteria have been revised, and avoidance and emotional numbing symptoms have been grouped into two distinct clusters^2^. This study showed that only the avoidance symptom cluster, not the emotional numbing symptom cluster, was associated with NoGo-P3 latency, which provides electrophysiological support for the DSM-5's current distinction between avoidance and emotional numbing PTSD symptoms.

The current results were from a sample of adolescent students. Adolescence is a period of increased vulnerability to psychiatric problems because of both the greater risk of experiencing trauma and incomplete maturation of the frontal lobe^18^,^19^. Therefore, the association between the avoidance scores and NoGo-P3 latency in adolescent earthquake survivors may have specific implications for developmental psychopathology. For example, this result may explain some of the potential behavioral difficulties associated with adolescent PTSD, such as behavioral control deficiency and risk-taking activities^50^.

This study had several limitations. First, a self-report measure, the PCL, was used to evaluate PTSD symptoms. Additional studies using clinically administered instruments are therefore needed. Second, the design of the present study does not allow any conclusions about causality in the observed association between the ERP measure and PTSD symptom severity to be drawn. Effortful avoidance may delay a later stage of response inhibition, but abnormal cognitive function, such as response inhibition, may increase the risk or severity of PTSD symptoms. Third, the results of this study may be explained by comorbid disorders. Participants in this study were not formally examined for comorbid disorders, although none of our subjects reported that they had suffered from neurological or major mental disorders. To exclude the possible effects of these clinical variables, we also used the HSCL-25 to assess participants' anxiety and depression score and performed a preliminary correlation analysis between these clinical symptoms and the behavioral/ERP measures before performing further regression analyses. Fourth, we did not measure the participants' intelligence quotient (IQ), a variable that could account for the difficulties in response inhibition and the likelihood of developing PTSD symptoms^51^. However, all of the participants were students from the same school, and all of them had passed the same entrance requirements for enrolling in the school, which suggests that these students have commensurate IQs and minimizes the likelihood that the observed correlations can be explained by intelligence-related factors. Finally, the generalizability of the current findings may also be limited by our utilization of a relatively small adolescence sample who suffered from a deadly earthquake. Therefore, additional replications with larger samples exposed to various traumatic events are warranted.

In conclusion, our clinical–electrophysiological correlation results suggest that avoidance symptoms, but not numbing or other symptom clusters, are associated with increased latency in the late step of response inhibition processing, i.e., the decision or success of response inhibition in adolescent earthquake survivors, providing biological support for the DSM-5's current distinction between avoidance and emotional numbing PTSD symptoms and helping to explain some of the potential behavioral difficulties associated with adolescent PTSD.

## Methods

### Participants

Participants were recruited through advertisements posted at Beichuan Vocational High School. The participants selected were those who had been directly exposed to the devastating earthquake in Wenchuan County, Sichuan Province, China, on May 12, 2008. We excluded students with (1) a past or current head injury; (2) self-reported neurological or major mental disorders, alcohol use (more than two alcoholic drinks daily or any alcohol use two days before the experiment), or substance use; or (3) those who had received psychiatric treatment or medication following the earthquake. Fifty-seven preliminarily qualified students expressed interest in participating. After determining which volunteers met the inclusion and exclusion standards, 54 students were selected (36 male) to participate. They ranged in age from 15 to 18 years (mean: 16.26 ± 0.96). All of them indicated via self-report that they were right-handed. All participants gave written informed consent and were paid for their participation. The experiment was approved by the Ethics Committee of Human Experimentation at the Institute of Psychology, Chinese Academy of Sciences. The methods were carried out in accordance with relevant guidelines and regulations.

### Questionnaires

The PTSD Checklist-Specific Stressor Version (PCL-S) developed by Weathers and colleagues^52^ was used to assess PTSD symptoms. The PCL is a 17-item self-report scale based on the PTSD symptoms described in the DSM-IV. In the PCL-S, respondents rate, from 1 (not at all) to 5 (extremely), the degree to which a particular symptom has bothered them during the previous month. The PCL is one of the most commonly used PTSD instruments, and its reliability and validity have been confirmed by several psychometric studies^43^,^53^. The Chinese version of the PCL was adapted from the English version following a translation and back-translation process, and its psychometric properties have been well documented^42^,^54^,^55^. In this study, participants were instructed to complete the PCL-S with reference to the Wenchuan earthquake.

The intensity of each participant's earthquake-specific trauma exposure was assessed by asking (1) whether the participant had been trapped as a result of the earthquake; (2) whether the participant had been physically injured by the earthquake; (3) whether the participant felt fear when the earthquake occurred; (4) whether one or more of the participant's family members had died because of the earthquake; (5) whether the participant witnessed others die in the disaster; and (6) whether the participant witnessed or touched a dead body during the disaster. The participants answered “yes” or “no” on a sheet of paper. The trauma exposure score was calculated by adding the scores of each item (no: 0; yes: 1).

The Hopkins Symptom Checklist-25 (HSCL-25) was used to evaluate the anxiety and depression level. It consists of 25 items: 10 items for anxiety and 15 items for depression, with scores ranging from 1 (not at all) to 4 (extremely)^56^. The participants reported on the symptoms they experienced up to one week before the survey was undertaken. The Chinese version of the HSCL-25 was revised through a translation/back-translation process by authors who were fluent in both English and Chinese^57^.

### Stimuli

The stimuli (either the digit “1” or “9”) were presented in white on a black background in the center of a computer screen at a visual angle of approximately 1.8° horizontally and 2.6° vertically.

### Procedure

The experiments were performed approximately 13 months after the earthquake. After completing the questionnaires, the participants were seated comfortably in a normally lit room. After a practice block of 18 trials, two experimental blocks, each consisting of 72 stimuli (50% NoGo and 50% Go), were completed, with the participants receiving a short break between the blocks. The equiprobability of the Go/NoGo design prevents the confounding of stimuli probability differences^58^,^59^. During each trial, one of the two stimuli was presented for 50 ms, followed by a random interstimulus interval of 1000–1300 ms. The participants were required to either give a response (Go) or withhold a response (NoGo) by pressing a button as accurately and as quickly as possible when a Go stimulus was presented but not when a NoGo stimulus was presented. The pairing of stimuli and Go/NoGo responses was counterbalanced across participants.

### EEG Recording and Preprocessing

In both experimental blocks, an electroencephalogram (EEG) was recorded from 64 scalp sites using Ag/AgCl electrodes mounted in an elastic cap (Compumedics Neuroscan, Charlotte, NC). The EEG had an online reference to the left mastoid and an offline algebraic reference to the average of the left and right mastoids. The vertical and horizontal electrooculograms were recorded from two pairs of electrodes. One pair was placed above and below the left eye, and the other, 10 mm from the outer canthi of each eye. Interelectrode impedance was maintained at <5 kΩ. The signals were amplified with a 0.05–100 Hz bandpass filter and digitized at 500 Hz.

The EEG data were digitally filtered using a 30-Hz low-pass filter and were epoched into periods of 700 ms (including a 100 ms prestimulus baseline) time-locked to the onset of the presented digit. Ocular artifacts were removed from the EEG signal using a regression procedure available through NeuroScan software. Trials with various artifacts were rejected if they exceeded the criterion of ±70 μV. The ERPs from both the Go and NoGo conditions were then averaged. Behaviorally incorrect trials were not included in the ERP averages.

The peak amplitudes and latencies of the frontocentral N2 and frontocentral P3 were measured at the Fz, FCz, and Cz sites. The peak amplitudes and latencies of the frontocentral N2—the minimum voltage between 200 and 400 ms poststimulus at each electrode—and the frontocentral P3—the maximum voltage between 300 and 500 ms—were measured for both the Go and NoGo conditions. The time windows were chosen based on the grand average ERP for each experimental condition.

### Data Analysis

All statistical analyses were conducted using SPSS software (version 19.0). Descriptive statistics were gathered from the questionnaire scores and behavioral data, including the reaction time in correct trials, the rate of omission errors in the Go trials, and the rate of commission errors in the NoGo trials. To examine the NoGo-N2/P3 effects, a repeated-measures analysis of variance (ANOVA) was conducted on the Go-NoGo factors and the amplitudes recorded at each of the three measurement sites (FZ, FCz, and Cz). The Greenhouse–Geisser correction was used to adjust for sphericity violations.

Bivariate associations between the severity of total PTSD symptoms and each of the behavioral and ERP measures (amplitude and latency averaged across the three measurement sites) were evaluated using regression analyses. Simultaneous multivariate regression analyses were conducted to examine the associations between the five PTSD symptom clusters and response inhibition. The symptom clusters were treated as predictors, and each of the behavioral and ERP measures of response inhibition were treated as dependent variables. All *p* values below 0.05 were considered statistically significant, and the tests were two-tailed. A preliminary correlation analysis was conducted to examine whether demographic variables (i.e., age and gender) and clinical variables (i.e., trauma exposure, depression, and anxiety) were significantly correlated with each of the dependent variables. The variables that had a significant association with the dependent variables were treated as covariates in the regression analyses.

## Author Contributions

Author J.W. wrote the first draft of the manuscript. Author Y.Y. and Author C.C. undertook the EEG data processing and statistical analysis. Author K.Z. designed the study and revised the draft. Author L.W. interpreted the results and revised the draft. Author L.Z. supervised the project, designed the study and analyzed the data. All authors contributed to and have approved the final manuscript.

## Figures and Tables

**Table 1:** Descriptive Statistics for the Behavioral Outcomes and PCL Scores (n = 54)

	Behavioral Outcomes			PCL Scores					
	CE (%)*	OE (%)*	RT (ms)*	PCL (Total)	RE*	AV*	EN*	DA*	AA*
**Mean**	7.20	5.48	354.83	39.63	11.57	4.33	10.28	8.44	5.00
**S.D.**	6.10	9.75	36.05	12.24	3.88	1.49	3.67	3.27	1.92
**Range**	0–31.94	0–45.83	251.08–425.76	19–73	5–22	2–8	5–20	3–15	2–10

Note: CE = rate of commission errors in NoGo trials; OE = rate of omission errors in Go trials; RT = reaction time in the Go trial; RE = re-experiencing; AV = avoidance; EN = emotional numbing; DA = dysphoric arousal; AA = anxious arousal; S.D. = standard deviation.

**Table 2:** The relationship between the PTSD symptom clusters and NoGo-P3 latency (averaged across Fz, FCz, and Cz) (n = 54), and the collinearity statistics of the multivariate regression model

Symptom cluster	r	B	β	t	p	Tolerance	VIF
**Re-experiencing**	0.140	−0.350	−0.041	−0.159	0.874	0.257	3.890
**Avoidance**	0.362	9.427	0.428	2.699	0.010	0.692	1.446
**Emotional numbing**	0.058	−1.120	−0.125	−0.648	0.520	0.467	2.139
**Dsyphoric arousal**	0.058	−2.043	−0.204	−0.804	0.425	0.272	3.675
**Anxious arousal**	0.139	3.991	0.234	0.868	0.390	0.240	4.163

Note: VIF = variance inflation factors.
